# Efficient isolation and purification of tissue-specific protoplasts from tea plants (*Camellia sinensis* (L.) O. Kuntze)

**DOI:** 10.1186/s13007-021-00783-w

**Published:** 2021-07-29

**Authors:** Xue-feng Xu, Hai-yan Zhu, Yin-feng Ren, Can Feng, Zhi-hao Ye, Hui-mei Cai, Xiao-chun Wan, Chuan-yi Peng

**Affiliations:** 1 State Key Laboratory of Tea Plant Biology and Utilization, Anhui Agricultural University, Hefei, 230036 Anhui People’s Republic of China; 2 Key Laboratory of Food Nutrition and Safety, School of Tea and Food Science & Technology, Anhui Agricultural University, Hefei, 230036 Anhui People’s Republic of China

**Keywords:** *Camellia sinensis* (L.) O. Kuntze, Protoplast, Isolation, Purification, Tissues

## Abstract

**Background:**

Plant protoplasts constitute unique single-cell systems that can be subjected to genomic, proteomic, and metabolomic analysis. An effective and sustainable method for preparing protoplasts from tea plants has yet to be established. The protoplasts were osmotically isolated, and the isolation and purification procedures were optimized. Various potential factors affecting protoplast preparation, including enzymatic composition and type, enzymatic hydrolysis duration, mannitol concentration in the enzyme solution, and iodixanol concentration, were evaluated.

**Results:**

The optimal conditions were 1.5% (w/v) cellulase and 0.4–0.6% (w/v) macerozyme in a solution containing 0.4 M mannitol, enzymatic hydrolysis over 10 h, and an iodixanol concentration of 65%. The highest protoplast yield was 3.27 × 10^6^ protoplasts g^−1^ fresh weight. As determined through fluorescein diacetate staining, maximal cell viability was 92.94%. The isolated protoplasts were round and regularly shaped without agglomeration, and they were less than 20 μm in diameter. Differences in preparation, with regard to yield and viability in the tissues (roots, branches, and leaves), cultivars, and cultivation method, were also observed.

**Conclusions:**

In summary, we reported on a simple, efficient method for preparing protoplasts of whole-organ tissue from tea plant. The findings are expected to contribute to the rapid development of tea plant biology.

## Introduction

Plant protoplasts, totipotent, viable cells from which the cell walls have been enzymatically or mechanically removed, are targeted for the fusion of exogenous nucleic acids and cell organelles [1, 2]. Plant protoplasts provide unique, single-cell systems for investigating the aspects of genomics, proteomics, and metabolomics [3]. With the rapid development of genome editing and gene silencing techniques, protoplasts have wide utility in technologies involving clustered regularly interspaced short palindromic repeats (CRISPR) and CRISPR-associated protein 9. Thus, the development of an efficient protoplast preparation method constitutes a profitable and worthwhile endeavor for research on the biology and physiology of the tea plant. Protocols for protoplast isolation and protoplast-based transient gene expression have been established for various herbaceous species, i.e*.*, *Arabidopsis thaliana* [4], *Zea mays* L. [5], as well as for woody species, i.e., the pomelo and tangerine [6], and the domesticated apple [7]. The tea plant, *Camellia sinensis* (L.) O. Kuntze, which is perennial and woody, is an economically valuable crop that is rich in theanine, tea polyphenols, and tea polysaccharides. The numerous health benefits of tea and its derivative products are well documented [8, 9]. In the literature on *C. sinensis*, the main focus is on the development of protoplast-derived transgenic plants. Progress is slow; functional gene verification has only been completed for heterologous chromosomes of *Arabidopsis thaliana* and *Nicotiana tabacum* L. [10, 11]. It is acknowledged that the lack of effective and sustainable methods for preparing tea plant protoplasts have seriously limited the development of tea biology. Notably, poor results have been reported for protoplast preparation from tea plants, as indicated by low efficiency, yield, and viability of specific tissues [12, 13]. A rapid, effective, and sustainable system for the isolation and purification of protoplasts from tea plants has yet to be established.

In the present study, we optimized procedures of protoplast isolation and purification for various tissues (i.e., roots, branches, and leaves) of tea plants. We investigated potential influencing factors of protoplast preparation: enzymatic composition and type, enzymatic hydrolysis duration, mannitol concentration, iodixanol concentration (purification effect), tea variety, and cultivation methods. To the best of our knowledge, this is the first report of the successful preparation of protoplasts from the entire tea plant. The proposed method may be useful in functional gene and stress physiology analysis and in breeding applications.

## Materials and methods

### Plant materials

#### Hydroponic branch cutting seedlings

**Fig. 1:**
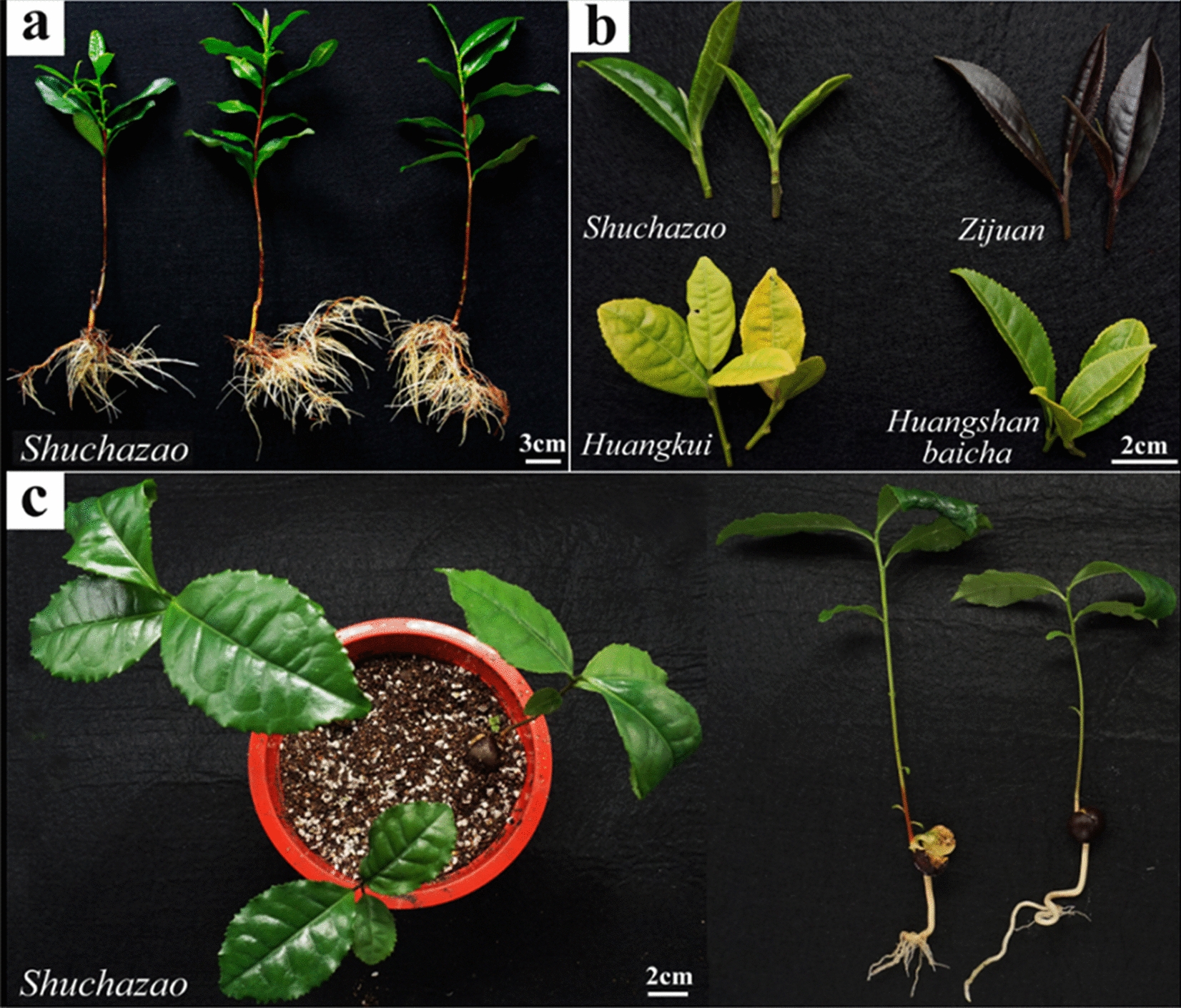
Plant materials. **a** Hydroponic cutting seedlings; **b** Tea plantation seedlings; **c** Potted seed seedlings

One-year-old branch cutting seedlings of *C. sinensis* var. *sinensis* cv. Shuchazao were obtained from Dechang Tea Fabrication Base in Anhui Province, China. Seedlings with uniform growth were selected and cultured hydroponically as we described previously [14, 15]. Afterwards, as shown in Fig. 1a, the newly expanded roots of the tea plants were picked for protoplast isolation and purification.

#### Natural tea plantation seedlings

For protoplast isolation and purification, unlignified branches, tender leaves (a bud and first leaf), and mature leaves (from the second to the fifth leaves) of Shuchanzao cultivar were selected (Fig. 1b); tender leaves (a bud and first leaf) were picked from tea plants belonging to the Huangshanbaicha, Zijuan, Huangkui, and Shuchanzao cultivars of *C. sinensis* var. *sinensis* grown on the campus plantation, from September to October 2020 (Fig. 1b).

#### Potted seedlings

The seeds of the Shuchazao cultivar were purchased from Wu Nong Trading Co., Ltd. (Xiaogan, Hubei Province, China). After cleaning and sterilization, three or four seeds were placed in a plastic pot with nutritional soil. After 5-month cultivation (Fig. 1c), roots, unlignified branches, and tender leaves were picked for protoplast isolation and purification, against the tissues from natural tea plantations (Fig. 1b).

### Enzyme solution preparation

The enzyme solutions were freshly prepared through enzymatic treatment. Regarding the optimal conditions for protoplast isolation, a mixture of macerozyme R-10 (Yakult, Japan) and cellulase R-10 (Yakult, Japan) was used. In brief, 20 mM 2-ethanesulfonic acid (pH = 5.7), 0.4 M mannitol, 20 mM KCl, 10 mM CaCl_2_, and 0.1% bovine serum albumin were dissolved in sterilized deionized water containing the enzymes, passed through a 0.45 μm syringe filter for sterilization, and then distributed into tubes in 10 mL portions and stored at − 20 °C before enzymatic hydrolysis. In the preparation of the enzyme solutions, mannitol concentrations (0.3 M, 0.4 M, 0.5 M, and 0.6 M) were examined to provide a suitable osmotic pressure and thereby ensure the integrity of the protoplasts upon their release from the tissues under enzymatic treatment.

### Protoplast isolation

As mentioned, the protoplasts were osmotically isolated. The procedure was optimized from the protocols developed for other plants in previous studies [2, 16, 17]. The samples were surface sterilized by dipping into 75% (v/v) alcohol for 30 s, then approximately 1 g fresh weight (FW) samples were cut into thin strips (0.5–1 mm) under sterile conditions, hydrolyzed with 10 mL of enzyme solution under negative pressure (− 0.1 MPa) and at room temperature for 30 min, and then incubated at 25 °C under gentle shaking (45 rpm) for enzymolysis [17]. All the steps of the isolation procedure were conducted under dark and sterile conditions.

Potential factors affecting concentrations of cellulase R-10 (1.0%, 1.5%, 2.0% and 2.5%) and macerozyme R-10 (0.2%, 0.4%, 0.6% and 0.8%) in the enzyme solutions were investigated in tender leaves, as were pectolase concentrations (0.025%, 0.05%, 0.10%, 0.30%, 0.5% and 0.7%) and enzymatic hydrolysis durations (6 h, 8 h, 10 h, and 12 h) Furthermore, tea samples from different tissues (newly expanded roots, unlignified branches, tender leaves, and mature leaves), cultivars (a bud and first leaf from Huangshanbaicha, Zijuan, Huangkui, and Shuchanzao, respectively), and cultivation methods (natural tea plantations vs potting) were examined and compared.

### Protoplast purification

After enzymolysis, the enzyme mixture was passed through a 100 μm cell filter. The filtrate was centrifuged at 200×*g* for 3 min. The protoplast pellets were resuspended in W5 solution (2 mM MES, 154 mM NaCl, 125 mM CaCl_2_, 5 mM KCl; pH 5.7). The filtrate was centrifuged in 50 mL round-bottomed centrifugal tubes at 200×*g* for 3 min to sediment the protoplasts. A 1 mL aliquot of iodixanol (31%, 45%, or 65%) was used for stratification. The purified protoplasts were suspended in iodixanol; they appeared as a green layer, which was collected with a 1 mL syringe for analysis. The purification procedure was conducted at 4 °C.

### Protoplast yield calculation and viability assessment

Protoplast yield was determined using a double-chamber hemocytometer under an inverted fluorescence microscope (Olympus IX73, Japan), and protoplast viability was determined by staining with 0.01% (w/v) FDA [18]. Protoplasts were considered viable if they exhibited green fluorescence. Counts for each sample were performed in at least three fields. Protoplast yield was calculated as follows:

$$\begin{aligned} {\text{Protoplast}}\,{\text{yield}}\,[{\text{protoplast}}\,{\text{g}}^{{ - 1}} \,{\text{FW}}] = {\text{number}}\,{\text{of}}\,{\text{protoplasts}}\,{\text{yielded}} \\ {\text{in}}\,{\text{enzyme}}\,{\text{solution}}/{\text{FW}}\,{\text{of}}\,{\text{the}}\,{\text{plantlet}}\,{\text{samples}}\,{\text{used}}. \\ \end{aligned}$$

Protoplast viability was calculated as follows:

$${\text{Protoplast}}\,{\text{viability}}\,(\% ) = \left( {{\text{number}}\,{\text{of}}\,{\text{fluorescent}}\,{\text{protoplasts}}\,{\text{in}}\,{\text{view}}/{\text{number}}\,{\text{of}}\,{\text{total}}\,{\text{protoplats}}\,{\text{in}}\,{\text{view}}) \times 100\% } \right.$$

Each purified protoplast was subjected to yield calculation and viability assessment five times in random order. Each experiment was performed in triplicate.

### Statistical analysis

Analyses were conducted using IBM SPSS Statistics for Windows, version 22 (IBM Corp., Armonk, NY, USA). Differences between treatments were considered significant at *P* ≤ 0.05 or *P* ≤ 0.01 according to the least significant difference. Data are expressed as means ± standard errors of the mean from three independent experiments, and were graphed using Prism 5.0 (GraphPad Software, La Jolla, CA).

## Results and discussion

### Optimized procedures for protoplast isolation and purification

Protocols for the isolation and purification of protoplasts from tea leaves have been reported [12, 13]. However, the separation efficiency remains relatively low and experimental replicability is poor; moreover, these protocols do not apply to the preparation of protoplasts from the roots and branches of tea plants. In view of these considerations, the present procedures were evaluated and optimized to establish an efficient protocol for the isolation and purification of protoplasts from various parts of tea plants.

**Fig. 2:**
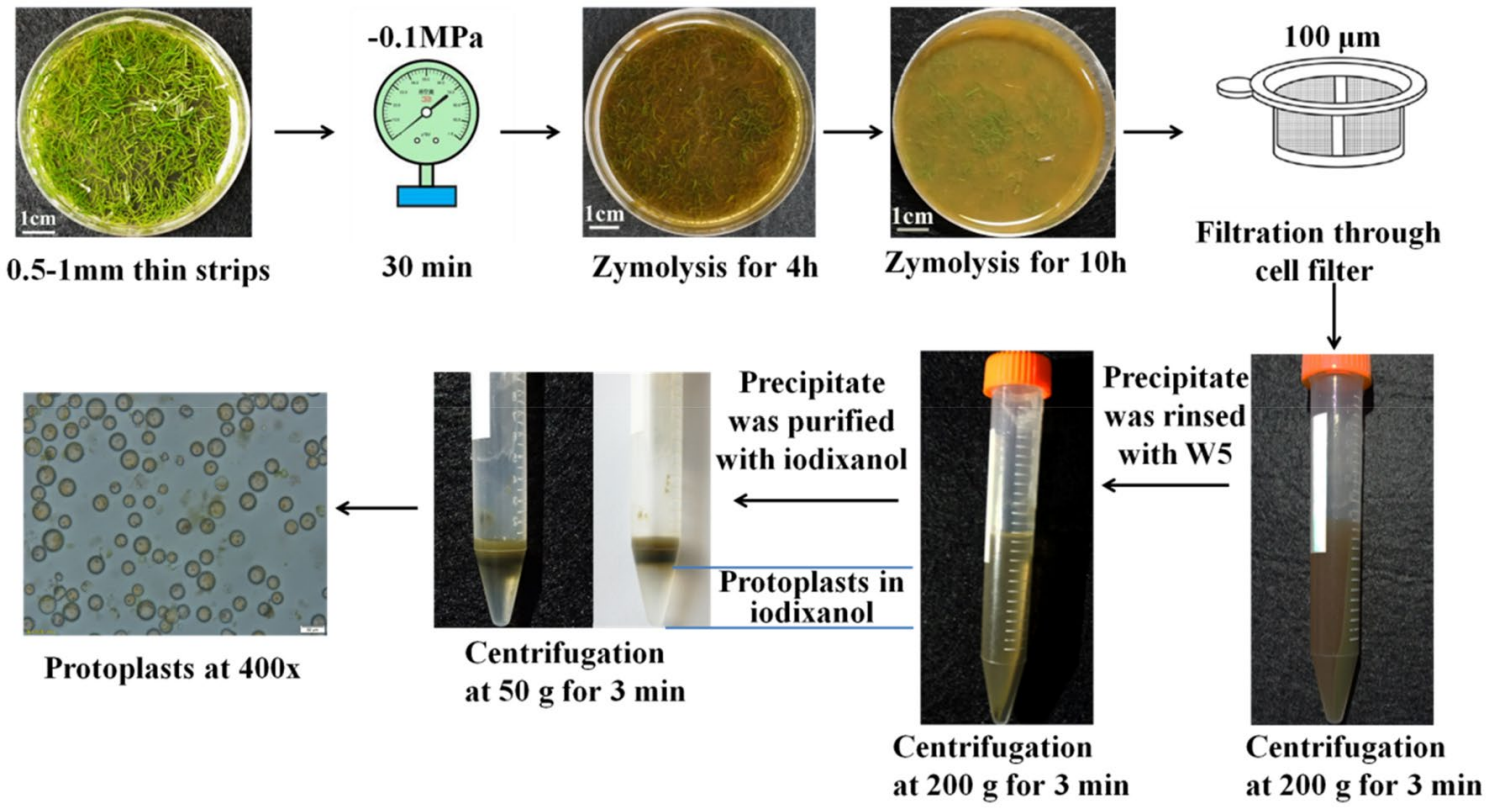
Optimized procedures of protoplast isolation and purification

The detailed procedures of protoplast isolation and purification were shown in Fig. 2, after vacuum assisted treatment and enzymolysis, cellulase–macerozyme hydrolyte was passed through a 100 μm cell filter and centrifuged at 200×*g* for 3 min. The green precipitate was rinsed with precooled W5 solution and then centrifuged at 200×*g* for 3 min once more to obtain the supernatant. The protoplasts were purified in precooled 65% iodixanol and then centrifuged at 50×*g* for 3 min. A clear boundary appeared, separating the protoplasts from the precipitate, and the protoplasts were assembled in the iodixanol layer at the bottom of the tube, clean and vibrant protoplasts were obtained. All purification operations were conducted at 4 °C. Compared the previous results from Liu et al.[12] and Peng et al.[13], the procedures of protoplast isolation and purification were optimized with vacuum assisted treatment for improving enzymolysis efficiency, and only once rinsing.

### Effects of enzymatic composition and type on protoplast isolation of tender leaves

**Fig. 3:**
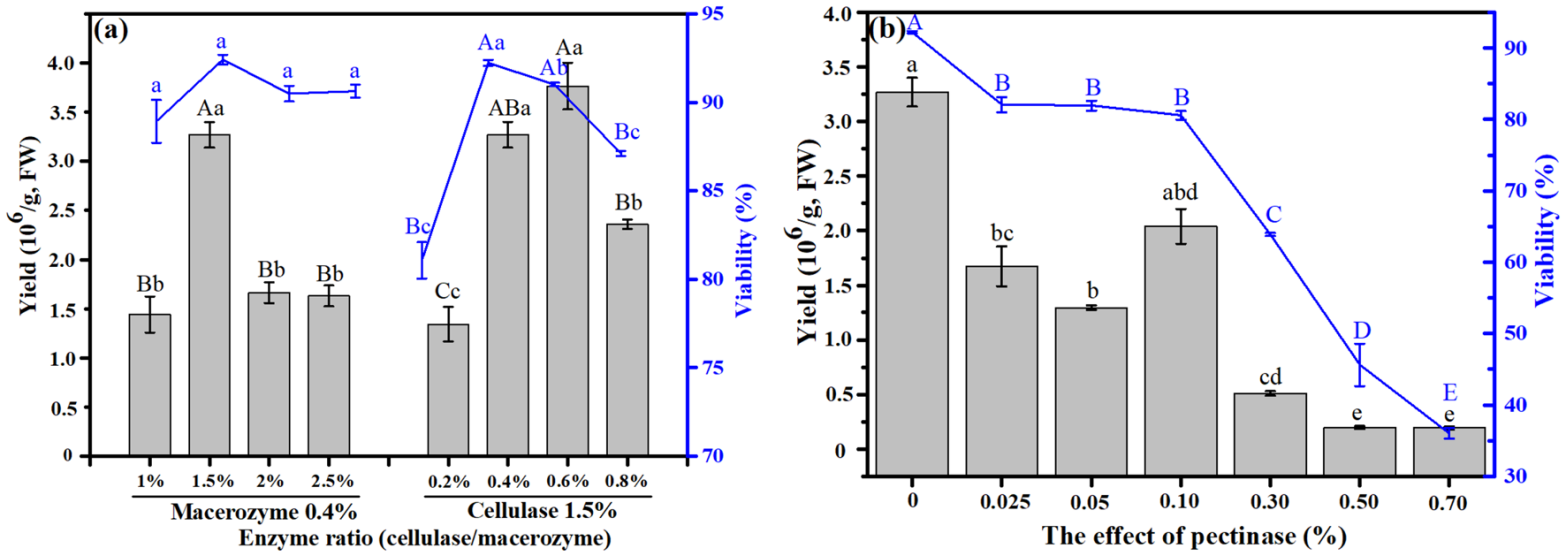
Effects of enzymatic composition (**a**) and type (**b**) on protoplast yield and viability. Different capital letters and small letters represent statistically significant differences at *P* < 0.05 and *P* < 0.01, respectively

To determine the optimal enzymatic composition and type, protoplasts were isolated from the tender leaves through various enzymatic digestion treatments for 10 h, and the protoplasts released under the appropriate osmotic pressure with 0.6 M mannitol concentration in the enzyme solution. The results of the enzymatic effects on the protoplasts are presented in Fig. 3. The effects of varying compositions of cellulase and macerozyme on protoplast yield and viability are shown in Fig. 3a. The highest yield of 3.27 × 10^6^ protoplasts g^−1^ FW was obtained when 1.5% cellulase was used. This yield was significantly higher than those obtained under other cellulase compositions. However, nonsignificant differences were observed for viability (*P* > 0.05). In terms of macerozyme effect, both protoplast yield and viability initially exhibited a downward trend and increased late in the process. Their peaks were 3.77 × 10^6^ g^−1^ FW and 92.24%, respectively. The yield and viability under 0.4% macerozyme and 0.6% macerozyme did not differ significantly (*P* > 0.01). Notably, pectolase negatively affected protoplast isolation (Fig. 3b) from response behaviors of the yield and viability, decreased from 3.27 × 10^6^ to 1.93 × 10^5^ g^−1^ FW and from 92.23 to 36.00%, respectively, as the amount of pectolase increases. In summary, yield and viability were optimized under 1.5% cellulase and 0.4–0.6% macerozyme.

#### Effects of enzymatic hydrolysis duration on protoplast isolation of tender leaves

**Fig. 4:**
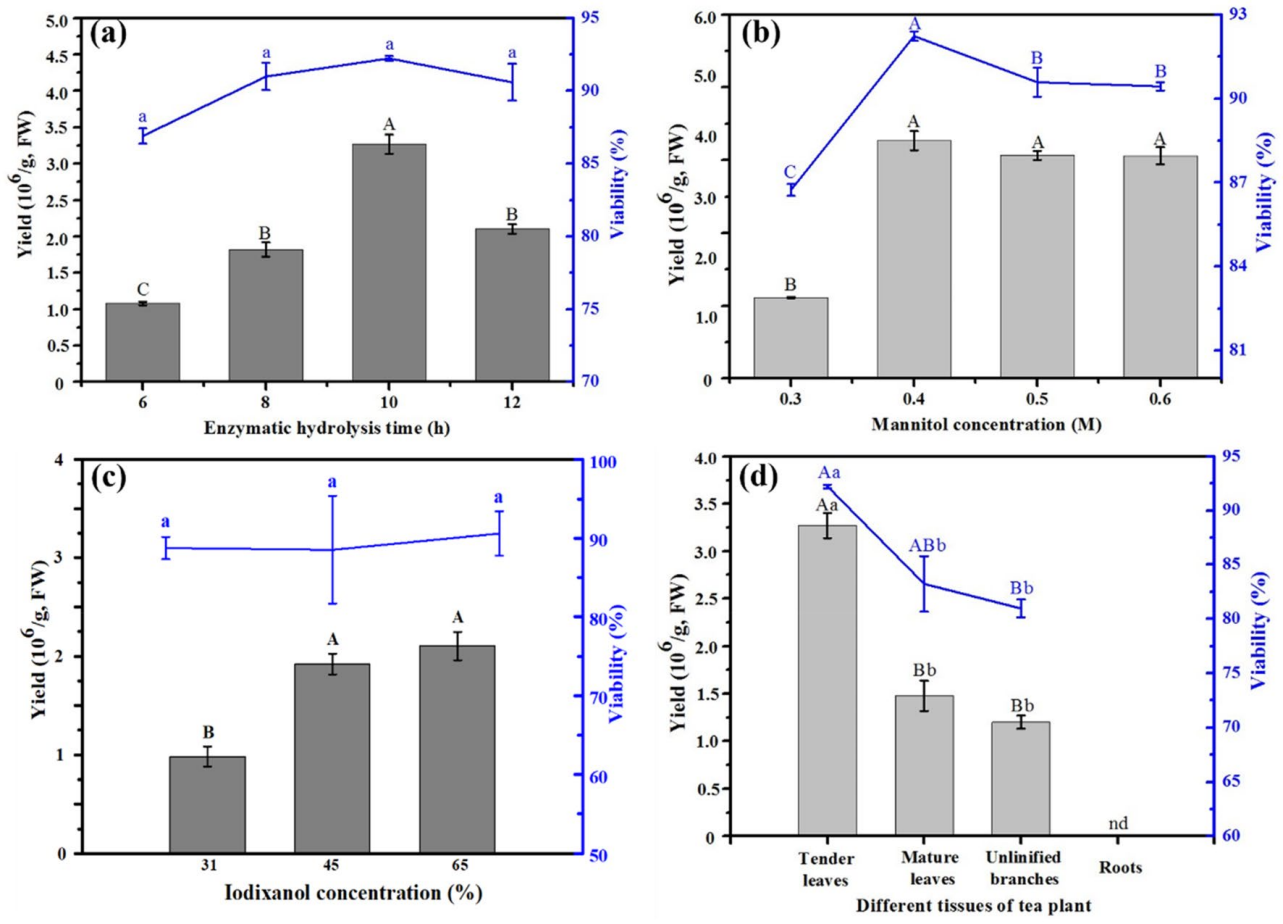
Effects of enzymatic hydrolysis duration (**a**), mannitol concentration (**b**), iodixanol concentration (**c**) and different tissue from hydroponic cutting seedlings (**d**) on protoplast isolation. nd indicates that the yield did not meet the counting requirements

After digestion with 1.5% cellulase and 0.4% macerozyme in a solution containing 0.4 M mannitol, the effects of enzymatic hydrolysis duration on protoplast isolation were assessed over the following durations: 6 h, 8 h, 10 h, and 12 h. As shown in Fig. 4a, viability did not differ significantly with duration (*P* > 0.05), but yield did (*P* < 0.01). Specifically, with duration, yield first increased (ranging from 1.08 × 10^6^ to 3.27 × 10^6^ protoplasts g^−1^ FW) and then decreased to 2.10 × 10^6^ protoplasts g^−1^ FW. The optimal duration was determined to be 10 h.

#### Effects of mannitol concentration on protoplast isolation of tender leaves

To optimize the concentration of mannitol in the enzyme solution to ensure the provision of appropriate osmotic pressure, experiments involving various concentrations of mannitol (0.3 M, 0.4 M, 0.5 M, and 0.6 M) were performed. The experimental duration was 10 h, and the cellulase and macerozyme concentrations were constant at 1.5% and 0.4%, respectively. Coupled with gradient changes of the mannitol concentration, protoplast yield and viability first increased (*P* < 0.01) and then reached equilibrium (Fig. 4b). The highest yield and viability of 3.27 × 10^6^ protoplasts g^−1^ FW and 92.24%, respectively, was obtained when 0.4 M mannitol was used. Yield did not increase significantly with further increases in mannitol concentration.

#### Effects of iodixanol concentration on protoplast purification of tender leaves

**Fig. 5:**
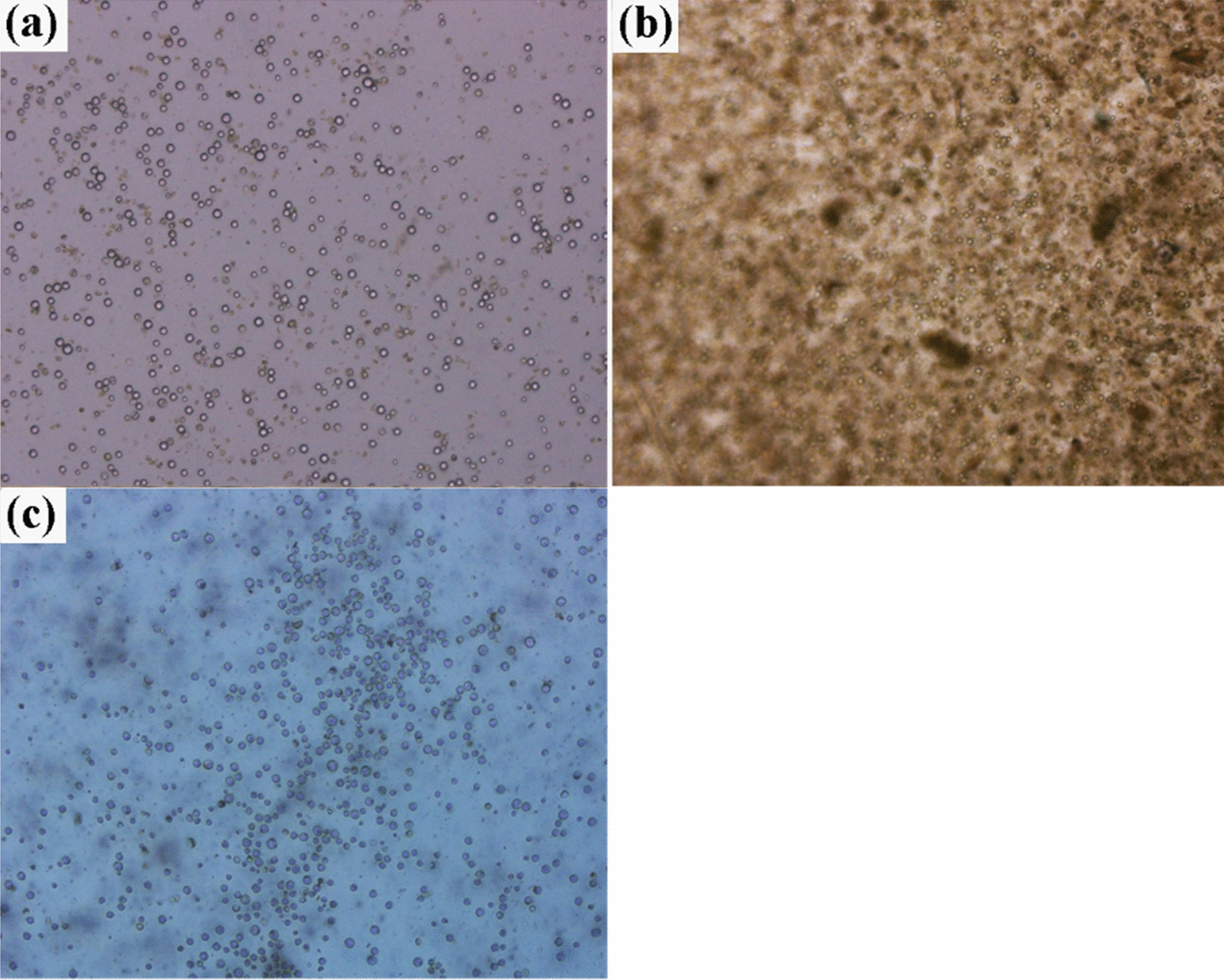
Effects of iodixanol and sucrose on protoplast purification. **a** 0.60 M sucrose; **b** 0.73 M sucrose; **c** 45% iodixanol

Sucrose is widely used to purify protoplasts obtained from purification many plant tissues [2, 19]. However, the present purification results were not very satisfactory (Fig. 5a, b). Sucrose with 0.6 M and 0.73 M could not completely separate the protoplasts from the impurities, especially when the sucrose concentration was 0.73 M (Fig. 5b). Iodixanol, a new, nonionic, dimeric contrast medium with low osmolality and osmolality used in computed tomography angiography, is widely applied in the isolation of animal cells [20, 21]. The objective of establishing the present method was to lay a foundation for the future examination of stress physiology response. Thus, an attempt was made to purify protoplast using iodixanol with effects evaluated for various concentrations (31%, 45%, and 65%). As shown in Fig. 4c, yields obtained under 45% and 65% iodixanol (1.92 × 10^6^ and 2.10 × 10^6^ g^−1^ FW, respectively) significantly exceeded those obtained under 31% iodixanol (9.83 × 10^5^ g^−1^ FW; *P* < 0.01), but viability did not differ significantly between these three concentrations (*P* > 0.05). Notably, the impurities observed under 65% iodixanol, as examined through microscopy, were significantly lower than those obtained under 45% iodixanol (Fig. 5c). Therefore, 65% was determined to be the optimal iodixanol concentration for purification.

In summary, protoplast yield and viability of tender leaves were optimized (3.27 × 10^6^ protoplasts g^−1^ FW and 92.24%, respectively) when 1.5% (w/v) cellulase and 0.4%–0.6% macerozyme were used in a solution containing 0.4 M mannitol assisted with negative pressure enzymolysis. The optimal relative centrifugal accelerations for rinsing and purification were 200×*g* and 50×*g*, respectively.

#### Effects of different tissues and growth conditions on protoplast preparation

**Fig. 6:**
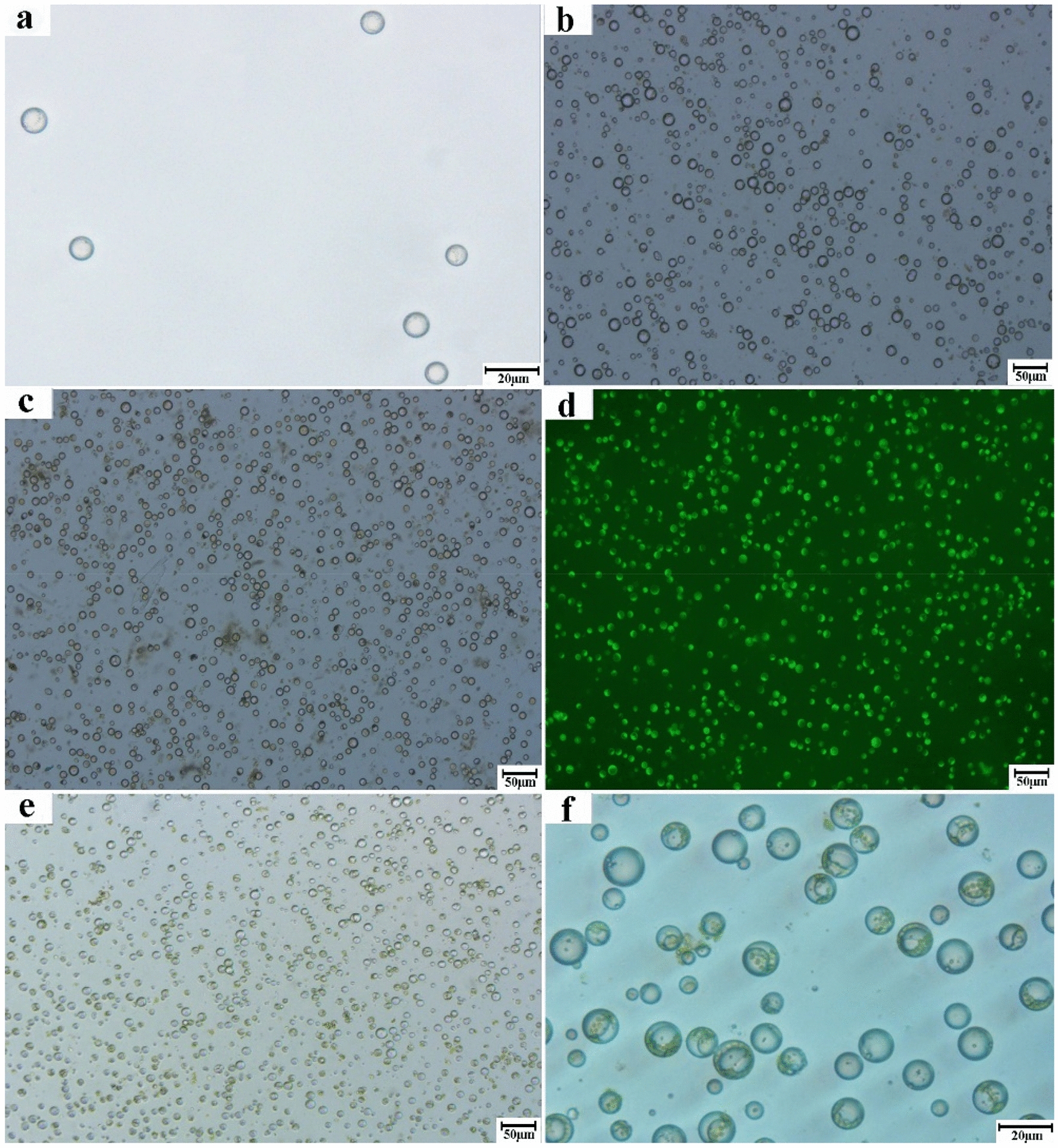
Effects of different tissue from hydroponic cutting seedlings on protoplast preparation. **a** roots (100×); **b** unlignified branches (100×); **c** tender leaves (100×); **d** tender leaves (fluorescence-labelled plant protoplasts, 100×); **e** mature leaves (100×); **f** mature leaves (400×)

**Fig. 7:**
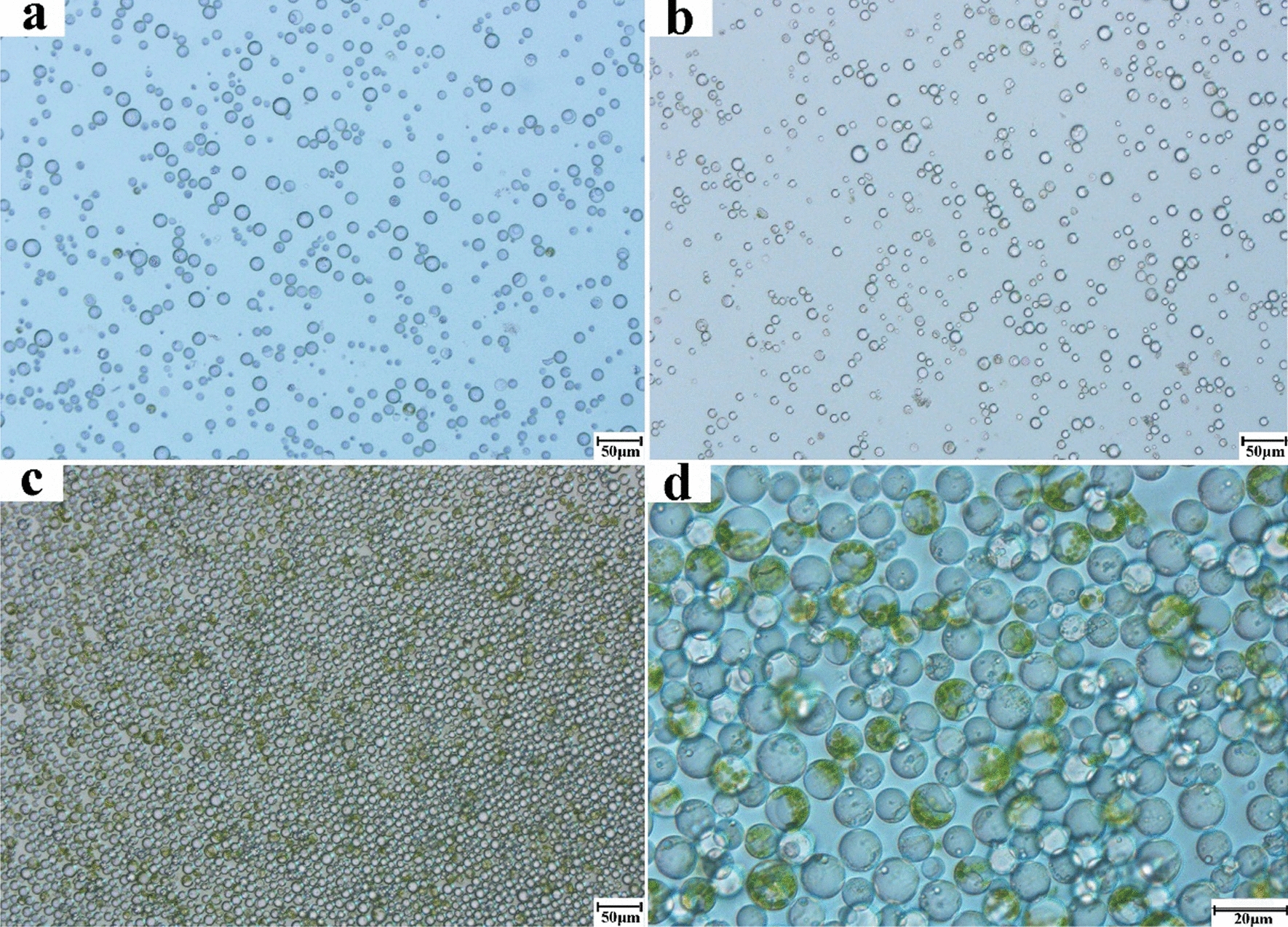
Effects of different tissue from potted cutting seedlings on protoplast preparation. **a** roots (100×); **b** unlignified branches (100×); **c** tender leaves (100×)

According to the previously optimized procedures and potential factors investigated, various tissues from the newly expanded roots, unlignified branches, tender leaves, and mature leaves of tea plants were also collected to establish an efficient procedure for protoplast preparation. The protoplast preparations of different tissues were shown in Fig. 4d, the yield and viability of protoplasts in the tender leaves were significantly higher than those of protoplasts in the mature leaves—2.21 times and 9% higher, respectively (*P* < 0.05, Fig. 4c and Fig. 6). By contrast, the yield and viability of the unlignified branches and the mature leaves did not differ significantly (*P* > 0.05). Failing to meet the counting requirements, only several holonomic protoplasts were obtained for the roots from the hydroponically grown cutting seedlings (Figs. 4c and 6a). The protoplasts were prepared more efficiently from the leaf base tissues than from the branches and roots. Nevertheless, protoplasts were efficiently prepared from the roots of the potted cutting seedlings (Figs. 1c and 7a), and the yield and viability of the other tissues also surpassed those of the samples from natural tea plantations (Figs. 6 and 7). This may be because tenderness is a key factor that influences protoplast preparation for tea plants, as indicated by previous results on the tender and mature leaves. Studies have demonstrated that young leaves, young embryos, calluses, and cell suspensions are the best materials for protoplast preparation, and that tissue culture seedlings were considerably better than potted or hydroponically grown cutting seedlings [6, 22]. Cultured seedlings are tender and sterile; moreover, their growing environment is easy to control and a wide range of stable sources are available providing a favorable foundation for protoplast preparation [22, 23].

Overall, the isolated protoplasts from the roots, branches, and leaves were less than 20 μm in diameter and were round and regularly shaped without agglomeration. Remarkably, the protoplasts isolated from leaves of the potted cutting seedlings were numerous and uniform in size (Fig. 7c, d). A large proportion of the protoplasts isolated from the tender and mature leaves were rich in chloroplasts (Figs. 6 and 7). The results are also notable in that this is the first time a substantial number of protoplasts with high viability has been isolated from tender branches of the tea plant.

#### Effects of different tea varieties on protoplast preparation

**Fig. 8:**
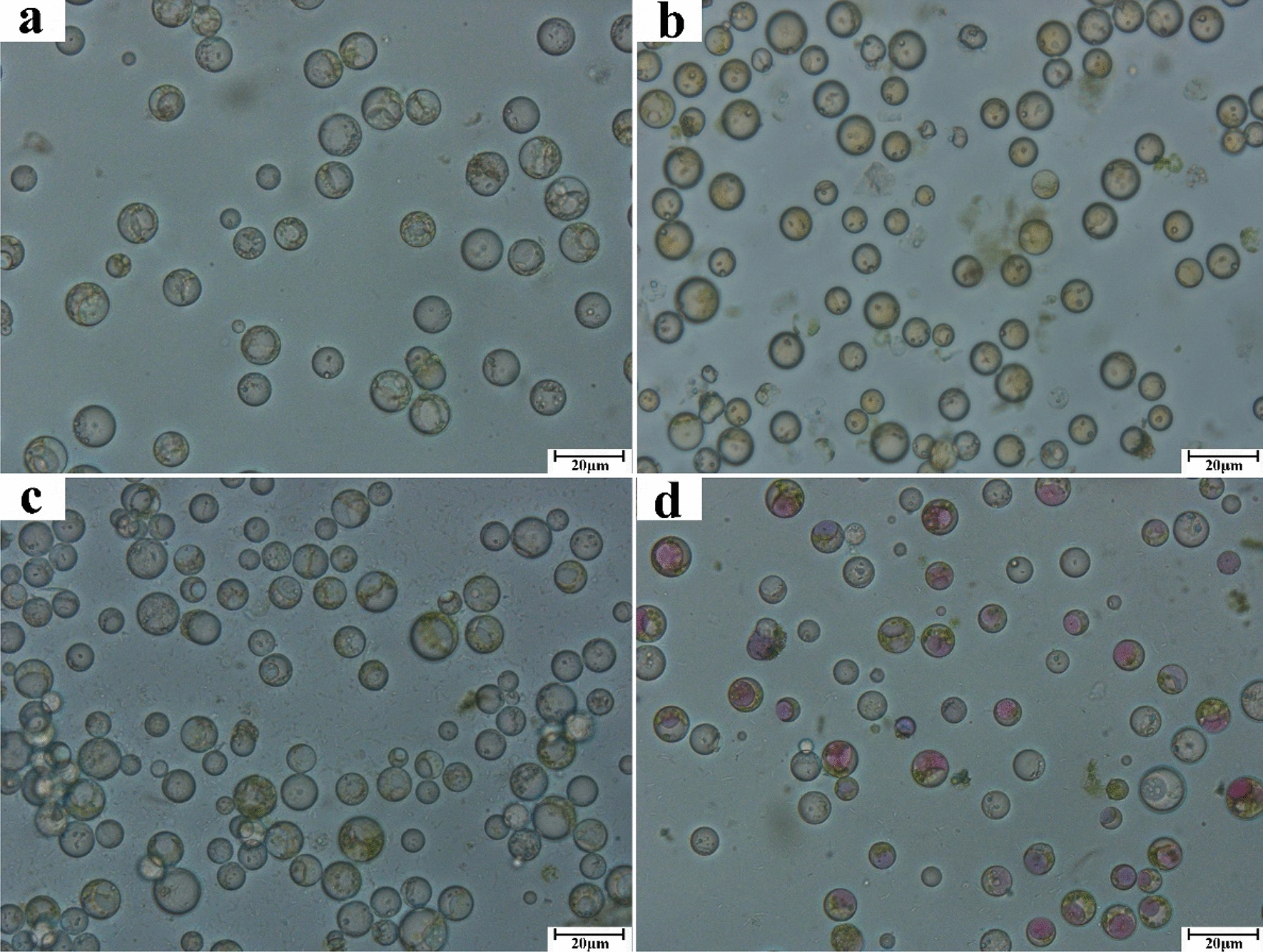
Effects of different tea variety on leaf protoplast preparation (400×). **a** Huangkui; **b** Shuchazao; **c** Huangshanbaicha; **d** Zijuan

**Fig. 9:**
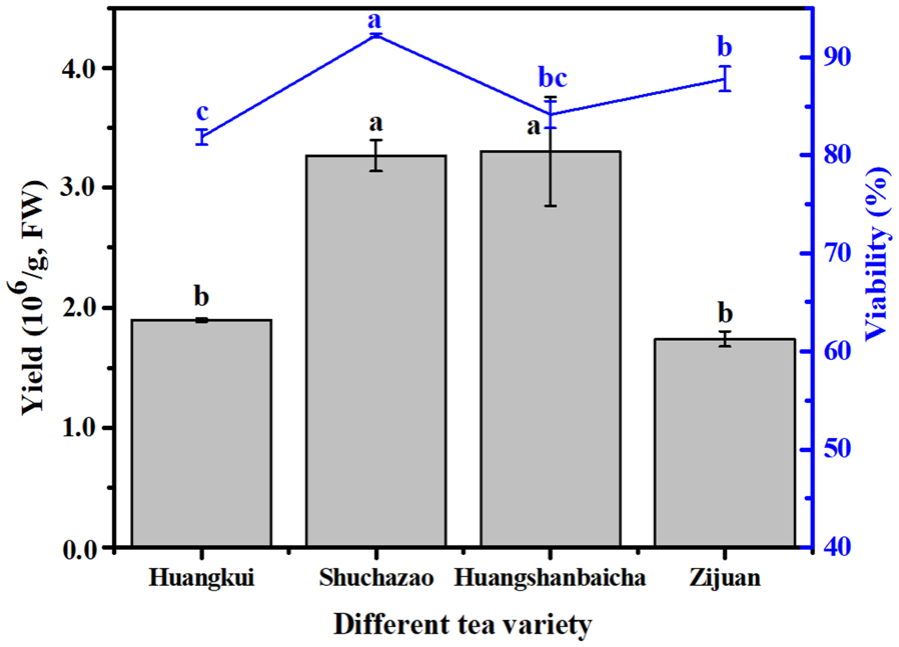
Effects of different cultivars on leaf protoplast preparation from plantation seedlings

To verify the applicability of the present optimization method, protoplasts were isolated from the tender leaves of the other three tea cultivars (Huangkui, Huangshanbaicha, and Zijuan) (Fig. 1b). As shown in Fig. 8, an abundance of pure protoplasts was obtained, and the anthocyanins were clearly observable. The protoplast yields from the Zijuan, Shuchazao, and Huangshanbaicha cultivars did not differ significantly (*P* > 0.05, Fig. 9), but protoplasts from the Shuchazao cultivar were significantly more viable (*P* < 0.05). The yields of the Huangkui and Zijuan cultivars were 1.89 × 10^6^ and 1.74 × 10^6^ protoplasts g^−1^ FW, respectively, and their viabilities were 81.86% and 87.80%, respectively. Thus, an efficient method of the isolation and purification of protoplasts from tea plants was established, as verified through the multiperspective analysis of the effects of variations in plant tissue and cultivar.

Table 1 presents a literature-based comparison of the efficiency of protoplast preparation under various efficient protoplast isolation protocols for woody and herbaceous plant species. The protoplast yields for most of the plants ranged from 10^5^ to 10^7^ g^−1^ FW, and protoplast viability was mostly higher than 80%. The viabilities of equiponderant protoplasts (10^6^ g^−1^ FW) obtained from the optimized protocol surpassed the results (50–89%) of tea leaves from Peng et al.[13], Liu et al. introduced the protoplast characteristics of first leaves through textual description without any data [13]. The method optimized in this study was effective and sustainable for isolating protoplasts from various tissues, including the roots, branches, and leaves, and from various tea species.

**Table 1:** Literature-based comparison of the isolation of protoplasts from various plants

Species	Tissue	Yield (g^−1^ FW)	Viability (%)	References
*Camellia sinensis* (L.) O. Kuntze	Tender leaves	3.27 × 10^6^	92.24	This study
	Mature leaves	1.48 × 10^6^	83.23	This study
	Unlignified branches	1.20 × 10^6^	80.97	This study
	Roots	3.20 × 10^6^	89	[13]
	First leaves	Description without data	Description without data	[12]
*Petunia hybrida*	Leaves	1.04 × 10^6^	73.3	[24]
*Ricinus communis* L.	Cotyledons and true leaves	6.1 × 10^6^	85	[25]
*Ananas comosus* L.	Leaves	6.5 × 10^5^	51	[26]
*Fragaria vesca*	Leaves	3.25 × 10^5^	–	[27]
*Saccharum spontaneum* L.	Young leaves	1.26 × 10^7^	–	[28]
*Chrysanthemum*	5–8-week-old leaves	6.32 × 10^5^	91.70	[29]
*Cymbidium*	Root	7.80 × 10^5^	89.3	[4]
	Flower pedicel	5.26 × 10^6^	90.3	
	Young leaf	3.30 × 10^6^	91.3	
	Leaf base	2.50 × 10^7^	92.1	
*Oryza sativa* L.	Stem and sheath tissues	1.00 × 10^7^	> 95.0	[30]
*Zea mays* L.	Middle parts (6–8 cm) of the second leaves	1.00–5.00 × 10^6^	95.0	[5]
*Manihot esculenta*	Fully expanded leaves	4.4 × 10^7^	92.6	[31]
*Arabidopsis thaliana*	Leaves	3.0 × 10^7^	–	[4]
*Malus Pumila Mill*	Cell suspension cultures	5.46 × 10^6^	98	[7]
	Cotyledon	3.72 × 10^6^	80	
	Leaves of the axenic shoot culture	3.57 × 10^6^	78	
	Leaves from garden	0.10 × 10^5^	32	
*Bamboo*	Leaves	> 6.67 × 10^6^	~ 83%	[32]
*Picea glauca (moench) voss*	Cells from 6-day-old subcultures	4.50 × 10^6^	70–90	[33]
*Pummelo* and *Tangerine*	Leaves	1.00–3.00 × 10^6^	91–96	[6]

## Conclusion

In this study, we obtained tissue-specific protoplasts from the root, branch, and leaf tissues of tea plants. In establishing the efficacy of the protocol, various factors affecting the efficiency of protoplast preparation, including enzymatic composition and type, enzymatic hydrolysis duration, mannitol concentration in the enzyme solution, and iodixanol concentration were evaluated. We also examined the tissue-, cultivar-, and cultivation-dependent differences in protoplast preparation. Our results indicated that the tender tissues at the leaf base of the tea plant are superior source materials for protoplast preparation, as indicated by the highest yield of viable protoplasts from these tissues. The application of the present optimization method could be expanded to other cultivars of *C. sinensis* and is expected to contribute crucially to functional genomic studies of tea plants.

## Data Availability

The datasets supporting the conclusions of this article are included within the article.
